# Phylogenetic position inferred on SSU rDNA sequence gene and description of a new parasitic cnidarian (Endocnidozoa: Myxobolidae) infecting *Markiana nigripinnis* (Teleostei: Stevardiinae) from a small marginal lake floodplain, Brazil

**DOI:** 10.22099/MBRC.2024.48723.1894

**Published:** 2024

**Authors:** Maria E.B.P. Mota, Patrik D. Mathews, Tiago Milanin, Omar Mertins, Fernando Paiva, Carina E. Oliveira, Luiz E.R. Tavares

**Affiliations:** 1 Department of Pathology, Institute of Bioscience, Federal University of Mato Grosso do Sul, 79070-900, Campo Grande, Brazil; 2 Department of Biodiversity and Biostatistics, Institute of Biosciences, São Paulo State University, 18618-689 Botucatu, Brazil; 3 Department of Basic Sciences, Faculty of Animal Science and Food Technology, University of São Paulo, 13635-900 Pirassununga, São Paulo, Brazil; 4 Laboratory of Nano Bio Materials, Department of Biophysics, Paulista Medical School, Federal University of São Paulo, 04023-062 São Paulo, Brazil; 5 Postgraduate Program in Biotechnology, Dom Bosco Catholic University, Campo Grande, Brazil

**Keywords:** Myxosporean, Gill parasites, Phylogeny, Tetra fish, Biodiversity

## Abstract

Herein, a detailed molecular phylogeny analysis was developed to determine the phylogenetic position of a new freshwater histozoic myxosporean cnidarian, *Henneguya markiana* sp. nov. from the world’s largest tropical wetland area, Pantanal, Brazil. The new species is described using an integrative taxonomy approach including morphology, biological traits and molecular data. Phylogenetic analysis inferred by Maximum Likehood method showed the new *Henneguya* species in a well-supported clade of myxosporean gill parasites of South American characids fishes. In this same clade, the new *Henneguya* described appeared in a sub-clade clustering with *H. lacustris* and *H. chydadea*. Nevertheless, the sequences of the new species and *H. lacustris* and *H.* *chydadea* have a large genetic divergence of 10.4% (148 nucleotides-nt) and 10.5% (147 nt) respectively. To the best of our knowledge, this is the first report of a cnidarian myxosporean species parasitizing a fish from Stevardiinae from South America. In the light of the differences observed from the integrative taxonomy, we are confident that this isolate is a new species of *Henneguya*, increasing the knowledge of diversity of this enigmatic group of cnidarians.

## INTRODUCTION

Floodplain environments are characterized by the existence of several aquatic and transitional habitats between those that are terrestrial and aquatic [1]. Among these diverse environments, marginal lakes of river-floodplain systems are widely recognized for their importance for maintaining integrity of fish diversity and regional biodiversity [2, 3]. Indeed, marginal lakes serve as nurseries for fish and/or constitute areas of growth and recovery of adult fish stocks [3]. At the same time, there is growing awareness of sensitivity and vulnerability to anthropogenic disturbances of theses aquatic habitats. Thus, fauna and flora knowledge that live in marginal lakes and their interactions are important for conservation strategies and polices in maintaining biodiversity [4]. In this context, this study describes a new freshwater cnidarian myxosporean and their interaction with the tetra fish *Markiana nigripinnis *Perugia, 1891 living in a floodplain small marginal lake from the Pantanal wetland biome, one the main biodiversity hotspots. 

Cnidarian myxosporean are an enigmatic group of eukaryotic organisms during many decades considered protozoans. They are obligate parasitic of worldwide distribution, which represent about 20% of presently known cnidarian biodiversity [5]. Myxosporean have a complex life cycle that diverged during the Cambrian era from free-living cnidarians [5, 6]. Among myxosporeans, members of the genus* Henneguya* Thelodan, 1892*, *are predominantly histozoic parasites recognized for their strict or high host and tissue specificity [7]. with some members being highly pathogenic for their host, causing severe henneguyosis [8-10]. Although South America contains one of the highest concentration of fish species living in a dynamic aquatic system composed of different freshwater habitats [11], there is scarce information about myxosporean cnidarian diversity. 

In this study, we provide detailed phenotypic and genotypic aspects of a new histozoic *Henneguya* cnidarian as well as their phylogenetic relationships and position among other myxobolids cnidarian members.

## MATERIALS AND METHODS

**Fish and Myxosporean collection: **A total of 58 specimens of *M. nigripinnis* (ranging from 6.5 to 8.2 cm in length) were collected by fishing net from a floodplain marginal lake (19°34.576´S, 57°,00.823´W), located in the sub-region of the Pantanal Miranda-Abobral, Municipality of Corumbá, State of Mato Grosso do Sul, Brazil. Fish were transported live in boxes containing dechlorinated water and artificial aeration to the Animal Parasitology Laboratory at Federal University of Mato Grosso do Sul, where they were housed in aquariums with dechlorinated water at a constant temperature of 27°C using thermostat systems (Hopar Aquarium Heater) and constant filtration and aeration (Aquatech FE25 Filtration System, prior to parasitological examination. Fish were euthanized in a benzocaine solution overdose (400 mg L^−1^) to carry out dissection all organs were examined under stereo and optical microscopes. Fish were identified according to Britski et al., [12] and current status such as valid species name or synonym were reviewed using FishBase [13]. Myxosporean cysts were carefully removed from the gills of fresh necropsied fish with aid of a Nikon SMZ1000 stereomicroscope and an Olympus BX53 microscope. 

**Morphological analysis: **Mature myxospores isolated from cysts, which were post-fixed in 10% formalin were used to performed morphometric analysis following the criteria outlined by Lom and Arthur [14]. Measurements of myxospores dimensions (spore length, thickness, polar capsule length, width, and caudal appendage length) were taken from 30 randomly selected mature fish, using a computer equipped with Axiovision 4.1 image capture software coupled to an Axioplan 2 Zeiss microscope (Carl Zeiss AG, Oberkochen, Germany). Myxospores dimensions were evaluated in micrometers (μm) and expressed as a mean ± standard deviation, followed by the range in parentheses where appropriate. Smears containing free myxospores were air-dried, fixed with methanol and stained with Giemsa stain to mount on permanent slides. These were deposited in the cnidarian collection of the Zoology Museum at the University of São Paulo – USP, São Paulo, Brazil (Hapantotype slide no. MZUSP 8695). Partial SSU rDNA sequence gene was deposited in GenBank under accession number (GenBank accession number: OR470748). The gill plasmodial index (GPI) was determined based on the criteria outlined by Kaur and Katock [15] and categorization of plasmodia on the basis of size follows Kaur and Attri [16]. 

**Molecular characterization, Genetic divergence and Phylogenetic analysis: **Genomic DNA (gDNA) was extracted from a cyst preserved in absolute ethanol using a DNeasy^®^ Blood & Tissue Kit (animal tissue protocol) (Qiagen Inc., California, USA), in accordance with the manufacturer's instructions. The gDNA concentration was measured using a NanoDrop 2000 spectrophotometer (Thermo Scientific, Wilmington, USA). Polymerase chain reactions (PCRs) were performed in accordance with Mathews et al., [17] and using primer routinely chosen for myxobolids molecular analysis (Table 1). PCRs were performed in the Mastercycler^®^ nexus (Eppendorf, Hamburg, Germany) and conducted in a final volume reaction of 25 μL, comprising 10–50 ng of extracted DNA, 0.2 pmol for each primer, 12.5 μL of Dream Taq Green PCR Master Mix (Thermo Scientific) and nuclease-free water. Thermal cycling amplification consisted of initial denaturation at 95°C for 5 min, followed by 35 cycles at 95°C for 60 s, 64°C (ERIB1, Barta et al., [18] -ACT1r, Hallet and Diamant [19]) or 58 °C (Myxgen4F, Diamant et al., [20]; ERIB10, Barta et al., [18]) for 60 s, 72°C for 120 s, and then final elongation at 72°C for 5 min. PCR products were electrophoretically under 2.0% agarose gel (BioAmerica, Irvine, California USA), in the presence of Sybr Safe DNA gel stain (Invitrogen by Life Technologies, Carlsbad, California USA) and analyzed with a Stratagene 2020E trans illuminator (Stratagene San Diego, California, USA). A1Kb Plus DNA weight ladder (Invitrogen by Life Technologies) was used to estimate of size of the amplicons. PCR products were purified using USB^®^ ExoSap-IT^®^ (Thermo Fisher Scientific) in accordance with manufacturer’s instructions. Sequencing was performed using the same PCR primers plus two additionally MC5 and MC3 primers [21] and using a BigDye^®^ Terminator v3.1 Cycle Sequencing kit (Applied Biosystems Inc., California, USA) in an ABI 3130 automatic DNA analyzer (Applied Biosystems Inc.™). Sequences obtained were assembled in BioEdit 7.1.3.0 [22]. An alignment search based on small subunit rDNA sequences (SSU rDNA) was performed to evaluate the similarity of newly sequenced and all sequences of myxobolids available in the GenBank [23]. Phylogenetic analysis was performed using Maximum likelihood (ML) and it was done in the PhyML 3.0 with Smart Model Selection [24]. Bootstrap analysis with 1000 replicates was employed to assess the robustness of the branches in the phylogenetic tree. Two South American freshwater myxosporean sequences, *Ceratomyxa amazonenses* KX236169 and *Ceratomyxa vermiformes* KX278420 were selected as outgroups. The genetic distance between *Henneguya*/*Myxobolus* species clustering together with the new sequence obtained was evaluate through pairwise comparison using MEGA 6.0 [25].

**Table 1:** Primers used in the amplification and sequencing of the SSU rDNA gene of *Henneguya markiana* sp. nov

**Primer**	**Sequences (5´–3´)**	**Reference**
**MC5**	CCTGAGAAACGGCTACCACATCCA	Molnar et al., [21]
**MC3**	GATTAGCCTGACAGATCACTCCACGA	Molnar et al., [21]
**ERIB10**	CTTCCGCAGGTTCACCTACGG	Barta et al., [18]
**Myxgen4F**	GTGCCTTGAATAAATCAGAG	Diamant et al., [20]
**ACT1r**	AATTTCACCTCTCGCTGCCA	Hallett and Diamant [19]
**ERIB1**	ACCTGGTTGATCCTGCCAG	Barta et al., [18]

## RESULTS

**Figure 1:**
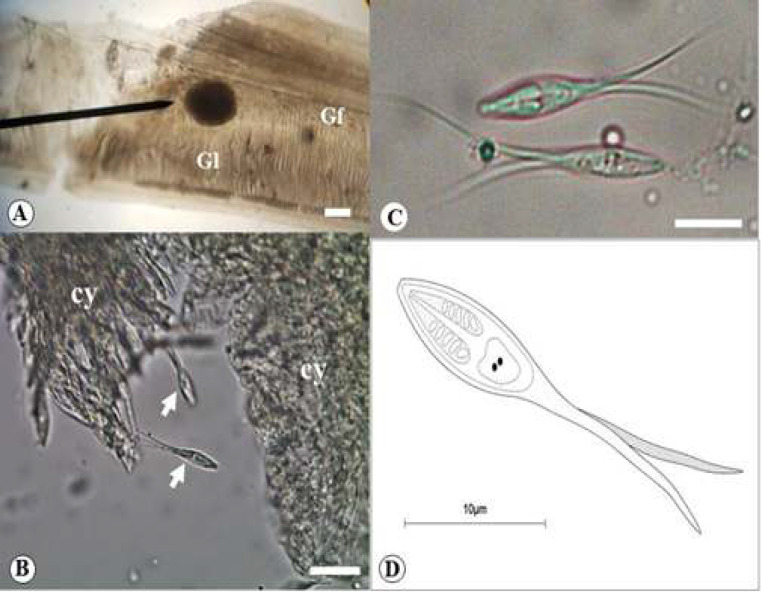
Gill lamellae infected with *Henneguya markiana* sp. nov. (A) Gill filament (Gf) showing a cyst (black arrow) in their lamellae (Gl). Scale bar: 30 µm (B) Unstained, compressed, ruptured cyst (cy), showing out myxospores (white arrows). Scale bar: 10 µm. (C) Mature myxospores in frontal view showing fusiform body with two equal polar capsules and bifurcate caudal appendage. Scale bar: 10 µm. (D) A schematic drawing of mature myxospore in ventral view

Out of 58 wild specimens of* M. nigripinnis* examined, 18 (31%) had gill lamellae infected by a new cnidarian myxosporean species of the genus *Henneguya* described herein. The specific name *Henneguya markiana* sp. nov. is based on host genus. The gill plasmodium index (GPI) correspondent to 1 (light infection) and category of plasmodium correspondent to Type A (visible under light microscope, size range 40–65 µm). Cysts were browned and rounded in shape, measuring 62.4 μm (58.3-64.1μm) in diameter (Fig. 1A). Mature myxospores were fusiform in shape from the frontal view, measuring 25.1 ± 0.6 μm (24.5–25.7 μm) in total length, 8.9 ± 0.4 μm (8.5-9.3 μm) in spore body length, 2.7 ± 0.2 μm (2.5 ± 2.9 μm) in with, 2.3 ± 0.4 μm (1.9 -2.7 μm) in thickness. Bifurcate caudal appendage, measuring 16.4 ± 0.4 μm (16–16.8 μm) in length (Fig. 1B). Two elongated polar capsules, occupying a little more than half the body, measuring, 4.6 ± 0.5 μm (4.1-5.1μm) in length and 1.9 ± 0.3 μm (1.6-2.2 μm) in with. Polar tubule had five coils (Fig. 1A-C).

Partial SSU rDNA gene sequence obtained of the newly *Henneguya* species resulted in 1434 nucleotides with a Cytosine-Guanine content of 49.6%. The BLASTn search (nucleotide Basic Local Alignment) revealed that the new sequence of this isolate did not match any other myxozoans available in GenBank and its sequence similarity with other *Henneguya *species was low (≤ 90%) with the highest similarity from *Henneguya lacustris *Mirandola, Rangel, Tagliavini, Abdallah, Santos, Azevedo, 2020 (query coverage 99%, maximum identities 89.8%), reported in the gills of *Astyanax lacustris *Lütken, 1875 from the Tietê river, State of São Paulo, Brazil [26]. Phylogenetic analysis inferred by ML method showed in the tree topology *Henneguya *spp. clustering with member of genus *Myxobolus*. The same ML tree evidenced a strong tendency among myxosporean species to cluster according to the taxonomic affinity of the fish. In the phylogenetic tree, the new sequence appeared in a clade of myxosporean gill parasites of South American characids fish. In this clade, the newly *Henneguya *sequence appeared in a sub-clade as the basal species cluster with *H. lacustris *and *H. chydadea *Barassa, Cordeiro, Arana, 2003, both parasites of the lambari-tambiú *A. lacustris* [26, 27]. Pairwise comparisons between the newly-obtained *Henneguya *sequence and the closest relatives resulted in genetic divergence of 10.4% (148 nucleotides-nt) to *Henneguya lacustris*, 10.5% (147 nt) to *H. chydadea*, 14.6 (207 nt) to *H. rotunda *Moreira, Adriano, Silva, Ceccarelli, Maia, 2014 and 14.3 (201 nt) to* Myxobolus pantanalis* Carriero, Adriano, Silva, Ceccarelli, Maia, 2013. 

## DISCUSSION

Although myxosporean represent about one fifth of the cnidarian biodiversity [5], there are biomes in many geographical areas for which there is a gap in the knowledge of myxosporean diversity [28-30], with the Pantanal wetland biome being a remarkable example. Indeed, from the Pantanal biome, a biodiversity hotspot harboring several potential host-fish, only four *Henneguya *species have been described to date [31, 32]. In the present study, we describe for the first time a myxosporean infecting fish of the subfamily Stevardiinae. We are also the first to report that these parasites infect fish from a genus *Markiana* from South America which increases our knowledge of the diversity of this group of parasite cnidarians.

Due to current discrepancies between the spore-morphology based classification and molecular taxonomy based on the small subunit ribosomal RNA [33-35], morphological traits are accompanied regularly by DNA sequences for description of new myxosporean species. At the same time, is highly recommended to integrate other non-DNA based characters for discriminating species such as the host and tissue infected [36]. Following the integrative taxonomy approach, morphological comparison was made considering all *Henneguya *species previously described to infect fish from the Pantanal wetland biome [32, 37], however, our comparison showed large numbers of noticeable morphometric differences as shown in Table 1. Furthermore, differences related to the host and tissue infected have been observed (Table 2). This result is in accordance with the high specificity of host and tissue, two biological characters widely recognized for freshwater histozoic myxobolids species [36]. The morphology of the new species described herein also was compared with closely related *Henneguya* spp. identified by a BLASTn search, however, noticeable morphologic differences were observed from these myxobolids (Table 2). 

**Table 2:** Comparative data of Henneguya markiana sp. nov. with all Henneguya spp. described in fishes from Pantanal wetland biome, including closely Henneguya species identified by a BLASTn search

**Species**	**TL**	**BL**	**APCL**	**SW**	**ST**	**PLC**	**PCW**	**NCT**	**Site of infection**	**Fish species**
***Henneguya markiana *** **sp.nov.**	25.1±0.6	8.9±0.4	16.4±0.4	2.7±0.2	2.3±0.4	4.6±0.5	1.9±0.3	5	Gill lamellae	*Markiana nigripinnis*
***Henneguya multiplasmodialis ***	30.8±1.3	14.7±0.5	15.4±1.3	5.2±0.3	4.4±0.1	6.1±0.1	1.4±0.1	6-7	Gill arch	*Pseudoplatystoma corrucans*
***Henneguya eirasi ***	37.1±1.8	12.9±0.8	24.6±2.2	3.4±0.3	3.1 ± 0.1	5.4±0.5	0.7±0.1	12-13	Gill filaments	*Pseudoplatystoma fasciatum*
***Henneguya corrucans***	27.6	14.3	13.7	5.0	-	6.8 (6–7)	2.0	5-6	Interlamellar space	*Pseudoplatystoma corrucans*
***Henneguya maculosus***	31.2	13.7±0.6	17.5±0.5	4.1±0.2	3.0±0.3	5.6±0.5	1.6±0.2	6-7	Gill filaments	*Pseudoplatystoma corrucans*
***Henneguya Chydadea***	17.6-20.0	8.8-11.2	8.0-9.6	3.2-5.6	-	3.2-4.4	1.2-1.6	9-10	Gill lamellae epithelium	*Astyanax lacustris*
***Henneguya lacustris***	18.3±2.2	10.4±1.6	7.2±2.5	4.9±0.9	-	4.8±0.3	1.5±0.2	6-8	Between the secondary lamellae	*Astyanax lacustris*
***Henneguya rotunda***	23.6±1.1	7.1±0.2	16.4±1.2	5.6±0.2	3.7± 0.1	3.4±0.2	1.8±0.1	6-7	Gill arch	Salminus brasiliensis

TL: total length; BL: body length; APCL: caudal appendage length; SW: spore width; ST: spore thickness; PCL: polar capsule length; PCW: polar capsule width; NCT: number of coils of polar tubules. Source: Rangel et al., [32], Eiras and Adriano [37].

**Figure 2:**
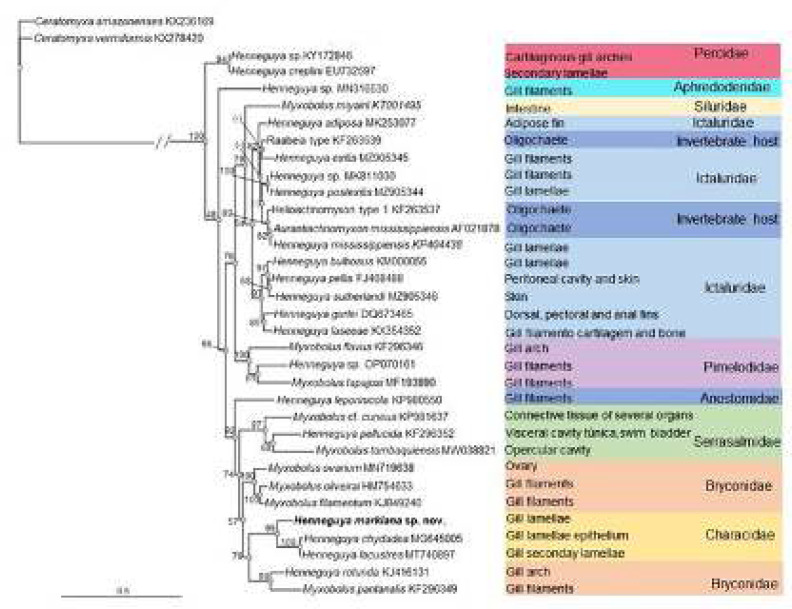
Maximum likelihood phylogenetic tree, based of SSU rDNA sequences of *Henneguya markiana* sp. nov. and other closely related myxobolid species based on BLAST. The tissue tropism and host taxonomy family are shown along the tree. The numbers above the nodes indicate bootstrap confidence levels. Bootstrap values below 40 are indicated in brackets. GenBank accession numbers are given for each species and in front of its

Performed molecular phylogenetic analysis shows *Henneguya* species clustering with *Myxobolus *species (Fig. 2). This result agrees with previous published phylogenetic studies based on analyses of ribosomal genes that demonstrated absence of phylogenetic separation between both genus [33,38,39] and they concluded that the caudal appendages of *Henneguya* spp. do not represent a valid character for a characterization of the genus. Although no clear line exists that separates *Henneguya *and *Myxobolus *genera, currently *Henneguya* is still valid taxonomically within myxosporean classification. Thus, we designated the isolated obtained herein as a new member of *Henneguya *genus based on their spore-morphology with presence of typical two caudal appendages [40]. Our phylogenetic analysis also showed (in all parts of the topology tree) strong relationships among *Henneguya* and *Myxobolus* species clustering in correlation with the fish taxonomic classification. This finding corroborates previous studies conducted in South America and other geographical regions [29, 38, 39, 41-43]. Conversely, our analysis also showed (in some clades in the phylogenetic tree, *Henneguya* and *Myxobolus* species) clustering according to their fish host tissue tropism, corroborating the observations offered by other authors [38, 39, 44]. This was largely noticeable in the clade from which the new sequence was obtained composed exclusively of myxobolid species that parasitize the gills of South American characids fish (Fig. 2). As pointed out by Carriero et. al. [38] tissue tropism has more evolutionary influence within clades that are formed by myxosporeans that infect fish hosts that are phylogenetically close. Thus, myxosporean species has tendency to clustered primarily by host affinity and in a second step, by the tissue infected. Since fish host group is a strong evolutionary signal within the Myxobolidae [38, 39], the correct host identification is highly recommended for description of new freshwater histozoic myxobolid species.

Finally, pairwise analysis showed a large genetic divergence between myxosporean that clustering in the same subclade with the new sequence obtained. This finding can be attributed to the host infected by these parasites with the new *Henneguya* from a Stevardiinae fish with the other species parasitizing fish belonging to the *Astyanax* genus. Indeed, previous studies have demonstrated by cytogenetic phylogeny as well as studies of sperm ultrastructure that *M. nigripinnis* (fish examined herein) is a very divergent species when compared with fish species from *Astyanax* genera [45-47]. It is important to point out that this is the first myxosporean sequence from a fish belong Stevardiinae. In the future, there is a need to sequence more myxobolid species from other fish taxa to demonstrate the accurate phylogenetic position. This will enable a better understanding about the evolutionary context of this new *Henneguya* species described herein.

### Acknowledgment:

M.E.B.P. Mota thanks CAPES for their MSc fellowship. P.D. Mathews thanks the São Paulo Research Foundation, FAPESP, for young researcher financial support (grant no. 2022/12376-4). L.E.R. Tavares was funded by CNPq (313292/2018-3, FUNDECT/CAPES: 59/ 300.134/2016). O.M. thanks CNPq for a research productivity grant. The authors are grateful to Kassia Capodifoglio, Antonio Maia and Márcia Ramos Monteiro da Silva for the support in laboratory analysis. The authors thank Dr. Christopher George Berger from Occidental College in Los Angeles, California for revision of the English language.

### Conflict of Interest:

The authors declare that no conflict of interest exists.

### Authors’ Contribution:

P.D.M., O.M. and T.M.: conceptualization, methodology, investigation, formal analysis, data curation, writing—original draft, revision. M.E.B.P. M.: collected and processed fish samples. F.P. and C.E.O: formal analysis. L.E.R. T.: Supervision. All authors have read and agreed to the published version of the manuscript.
